# Safety Profile of Low‐Power Pure‐Cut Hot Snare Polypectomy for 10–14 mm Nonpedunculated Colorectal Neoplasms

**DOI:** 10.1002/deo2.70235

**Published:** 2025-11-10

**Authors:** Kazunori Takada, Hidenori Kimura, Kinichi Hotta, Kenichiro Imai, Sayo Ito, Yoshihiro Kishida, Noboru Kawata, Masao Yoshida, Yoichi Yamamoto, Tatsunori Minamide, Hirotoshi Ishiwatari, Junya Sato, Hiroyuki Matsubayashi, Hiroyuki Ono

**Affiliations:** ^1^ Division of Endoscopy Shizuoka Cancer Center Shizuoka Japan; ^2^ Division of Digestive Endoscopy, Department of Medicine Shiga University of Medical Science Shiga Japan

**Keywords:** bleeding, colorectal neoplasms, electrocoagulation, hot snare polypectomy, adenomatous polyps

## Abstract

**Objectives:**

The reported rate of delayed bleeding (DB) after hot snare polypectomy (HSP) for 10–19‐mm polyps is 2.1%–2.8%, which is non‐negligible. We hypothesized that a low‐power pure‐cut current (LPPC) yields a lower risk of DB than a coagulation current, and we evaluated the safety of LPPC HSP for colorectal polyps.

**Methods:**

In this retrospective, observational study, consecutive patients who underwent LPPC HSP for nonpedunculated colorectal neoplasms sized 10–14 mm at two Japanese institutions from December 2018 to March 2022 were evaluated. We analyzed the treatment outcomes of LPPC HSP and compared the DB rate of LPPC HSP with that of the historical control of HSP, which was set as 2.1% based on a previous meta‐analysis.

**Results:**

A total of 339 patients (410 lesions sized 10–14 mm) were identified. The en bloc and R0 resection rates were 94.9% and 86.7%, respectively. Immediate bleeding requiring hemostasis developed in four lesions (1.0%). No perforations occurred. DB occurred in two patients; both had to be admitted but were conservatively managed without endoscopic hemostasis or blood transfusion. The DB rate was 0.6% for patients and 0.5% for lesions. LPPC HSP was associated with a 71.4% lower risk of DB than the historical control, with a power of 80.4% and a two‐sided significance level of 0.1.

**Conclusions:**

Considering its safety profile and resectability, LPPC HSP has the potential to supersede conventional resection methods. It may also be feasible for patients taking antithrombotic agents, who have a higher risk of DB.

**Abbreviations**
CI: confidence interval
CRC: colorectal cancer
CSP: cold snare polypectomy
DB: delayed bleeding
EMR: endoscopic mucosal resection
HSP: hot snare polypectomy
IQR: interquartile range
LPPC: low‐power pure‐cut current

## 1 Introduction

Endoscopic resection of colorectal adenomatous polyps can reduce the incidence of colorectal cancer (CRC) and related mortality [1]. Hot snare polypectomy (HSP) is the standard of care for the removal of nonpedunculated adenomatous polyps 10–19 mm in size [2]. The use of electrocautery induces thermal deep‐mural injury, resulting in delayed bleeding (DB). The reported DB rate after HSP for polyps 10–19 mm in size is 2.1%–2.8%, which is non‐negligible [3, 4]. A recent study reported the low incidence of invasive cancer to be 0.9% in this size category [5]. Thus, a less invasive removal method for polyps in this size category is desirable. Piecemeal cold snare polypectomy (CSP) can reduce the risk of DB, and multiple studies have shown its effectiveness for the removal of sessile serrated lesions [2]. However, the removal of adenomas via piecemeal CSP is associated with a high incidence of incomplete resection (21%) [6, 7] and recurrence (11%) [8], which can progress to post‐colonoscopy CRC [9]. The rate of en‐bloc resection via CSP for polyps 10–14 mm in size was unsatisfactory (82.5%) in one study, and 13.7% of those cases required a transition to HSP despite the administration of submucosal injections prior to CSP [10]. Therefore, cold excision is unsatisfactory, and en bloc HSP is the most reliable technique for adenomatous polyps 10–19 mm in size.

The en‐bloc resection rates of HSP and endoscopic mucosal resection (EMR) were similar for polyps 10–14 mm in size in randomized controlled studies, suggesting that submucosal injection for polyps in this size category is not mandatory [11, 12]. Interventions decreasing adverse events in HSP could develop a safer option in this size category. We hypothesized that eliminating the coagulation current with a lower voltage by using low‐power pure‐cut current (LPPC) can decrease the heat injury to the deeper tissue caused by HSP. In our experimental study, we showed that the remaining submucosal layer after HSP was thicker and that thermal damage to the muscular layer occurred less frequently with an LPPC than with a blended current [13]. Our phase I study of LPPC HSP for adenomas 10–14 mm in size displayed favorable resectability (complete resection: 85.7%, resection containing the submucosal layer: 88.8%) [13]. Although no DB was observed among the 90 patients with 100 polyps, the sample was insufficient to assess the safety of LPPC HSP. Therefore, we conducted a study aiming to evaluate the safety of LPPC HSP for colorectal adenomas.

## 2 Methods

### 2.1 Study Design and Patients

This retrospective, observational study included consecutive patients who underwent LPPC HSP for non‐pedunculated colorectal polyps 10–14 mm in size from December 2018 to March 2022 at two Japanese institutions: Shizuoka Cancer Center and Shiga University of Medical Science. Patients who participated in our phase I study were included in this analysis [13]. Following the confirmation of favorable results from that study, LPPC HSP became the predominant resection technique at the participating institutions. The exclusion criteria were as follows: 1) polyps diagnosed as type 1 according to the Japan Narrow‐Band Imaging Expert Team classification, suggesting that they are non‐adenomatous, and 2) patients with polyposis syndrome. Data were collected from medical records and prospectively recorded databases of endoscopic and pathological findings.

This study was approved by the Ethics Committee of Shizuoka Cancer Center (T2022‐56‐2022‐1). It was conducted in accordance with the Declaration of Helsinki and the Japanese Ethical Guidelines for Clinical Studies Involving Human Subjects. Informed consent was waived owing to the retrospective nature of the study, and participants could opt out of participation in research on the website of each institution. This manuscript was prepared according to the Strengthening the Reporting of Observational Studies in Epidemiology Statement [14].

### 2.2 Endoscopic Procedures of LPPC HSP

LPPC HSP was defined as a polypectomy performed without submucosal injection, using a low‐power pure‐cut current. LPPC HSP was performed using a 10‐ or 15‐mm thin hexagonal snare (SnareMaster Plus; Olympus Medical Systems, Tokyo, Japan) with either of the following modes: AutoCut effect 1, 10 W (VIO 300D; Erbe Elektromedizin GmbH, Tübingen, Germany), AutoCut effect 0.4 (Pmax 13 W; VIO 3; ERBE), or PureCut effect 1, 10 W (ESG‐300; Olympus) [13, 15]. High‐resolution colonoscopes with magnification functions (CF‐HQ290ZI, PCF‐H290ZI, CF‐EZ1500, or CF‐XZ1200; Olympus Medical Systems) were used for preoperative diagnoses of lesions based on white‐light imaging and image‐enhanced endoscopy, as well as for LPPC HSP. Lesions were measured with an endoscopic measurement device (M2‐3U; Olympus), forceps, or snares with predetermined diameters. Prophylactic clipping after resection was performed at the discretion of the endoscopist; however, as our phase I study reported no cases of DB [13], prophylactic clipping was generally deemed unnecessary. Endoscopic procedures were performed by 11 experienced endoscopists (certified by the Japan Gastroenterological Endoscopy Society with experience of >1500 colonoscopies and >100 polypectomies) and 15 gastrointestinal fellows (experience ≤1500 colonoscopies). The timing of stopping and restarting antithrombotic agents was based on the 2014 and 2017 guidelines of the Japan Gastroenterological Endoscopy Society [16, 17].

### 2.3 Outcome Measures and Definitions

The primary outcome measure was the DB rate, defined as the occurrence of bleeding necessitating either endoscopic hemostasis or hospital admission within 30 days of LPPC HSP [18]. Immediate bleeding was defined as intraprocedural bleeding requiring endoscopic hemostasis, such as spurting bleeding or continuous oozing that did not stop spontaneously within 30 s. En‐bloc resection was defined as endoscopic removal of the polyp in one piece with no visible neoplasms at the edge of the mucosal defect. R0 resection was defined as en bloc resection with no tumor tissue at the tumor margins upon histological analysis. The lesion location was divided into right‐sided colonic sections (cecum, ascending, and transverse colon), left‐sided colonic sections (descending and sigmoid colon), and rectum. Morphological assessment was performed according to the Paris classification [19]. Histological diagnoses were based on the Japanese classification of colorectal carcinoma [20].

### 2.4 Statistical Analysis

We compared the DB rate of LPPC HSP with that of historical controls of conventional HSP, which was set as 2.1% (55/2629) based on a previous meta‐analysis [4], and calculated the statistical power value by using a one‐sample proportion test. Continuous variables were expressed as medians and interquartile ranges. Categorical variables were expressed as frequencies and percentages. All statistical analyses were performed using EZR software (version 1.54; Saitama Medical Center, Jichi Medical University, Saitama, Japan) [21].

## 3 Results

### 3.1 Baseline Characteristics

**FIGURE 1:**
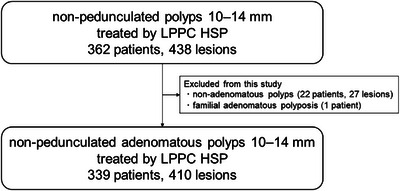
Study flow. LPPC HSP, low‐power pure‐cut current hot snare polypectomy.

A total of 339 patients with 410 lesions sized 10–14 mm were identified after excluding one patient with familial adenomatous polyposis and 22 patients with 27 lesions diagnosed as non‐adenomatous polyps (Figure 1). Of these, 316 patients with 379 lesions were from Shizuoka Cancer Center, and 23 patients with 31 lesions were from Shiga University of Medical Science. Patient and lesion characteristics are summarized in Table 1. Fifty‐five patients (16.2%) received antithrombotic therapy: 32 patients on a single antiplatelet, five on dual antiplatelets, 16 on anticoagulants, and two on anticoagulants and a single antiplatelet. The median lesion size was 11 mm.

**TABLE 1:** Patient and lesion characteristics.

	339 patients, 410 lesions
Age, years, median (IQR)	71 (67–77)
Male sex, *n* (%)	232 (68.4)
Antithrombotic agents, *n* (%)	55 (16.2)
Single antiplatelet	32 (9.4)
Dual antiplatelets	5 (1.5)
Anticoagulants	16 (4.7)
Anticoagulants and antiplatelet	2 (0.6)
Location, *n* (%)	
Right‐sided	235 (57.3)
Left‐sided	141 (34.4)
Rectum	34 (8.3)
Morphology, *n* (%)	
0‐IIa	189 (46.1)
0‐Is	133 (32.4)
0‐Isp	88 (21.5)
Lesion size, mm, median (IQR)	11 (10–12)
10 mm, *n* (%)	202 (49.3)
11 mm, *n* (%)	45 (11.0)
12 mm, *n* (%)	85 (20.7)
13 mm, *n* (%)	30 (7.3)
14 mm, *n* (%)	48 (11.7)
Performed by fellows, *n* (%)	81 (19.8)

Abbreviation: IQR, interquartile range.

### 3.2 Treatment Outcomes

The clinical outcomes of LPPC HSP are shown in Table 2. The en‐bloc and R0 resection rates were 94.9% (95% confidence interval [CI]: 92.3%–96.8%) and 86.7% (95% CI: 83.0%–89.8%), respectively. Four lesions were excluded from the assessment of R0 resection due to missing information on margin status. Horizontal margin was negative in 352 lesions (86.7%), indeterminate in 47 lesions (11.6%), and positive in seven lesions (1.7%). Vertical margin was negative in 404 lesions (99.5%) and indeterminate in two lesions (0.5%). Immediate bleeding requiring hemostasis occurred in four lesions (1.0%), each of which was successfully managed with endoscopic clipping. Prophylactic clipping was applied for 49 lesions (12.0%). No perforations occurred. Thirty‐one lesions were diagnosed as intramucosal cancer upon pathological analysis, 29 of which were resected with negative vertical margins. Four lesions were diagnosed as cancer with deep submucosal invasion, all of which were resected with negative vertical margins.

**TABLE 2:** Treatment outcomes.

	339 patients, 410 lesions
En‐bloc resection, *n* (%)	389/410 (94.9)
R0 resection, *n* (%)^a^	352/406 (86.7)
Horizontal margin, *n* (%)	
Negative	352/406 (86.7)
Indeterminate	47/406 (11.6)
Positive	7/406 (1.7)
Vertical margin, *n* (%)	
Negative	404/406 (99.5)
Indeterminate	2/406 (0.5)
Positive	0/406 (0)
Immediate bleeding, *n* (%)	4/410 (1.0)
Prophylactic clipping, *n* (%)	49/410 (12.0)
Perforation, n (%)	0 (0)
Delayed bleeding for patients, *n* (%)	2/339 (0.6)
Delayed bleeding for lesions, *n* (%)	2/410 (0.5)
Pathology, *n* (%)	
Adenoma	373 (91.0)
Traditional serrated adenoma	1 (0.2)
Sessile serrated lesion	1 (0.2)
Intramucosal cancer	31 (7.6)
Cancer with deep submucosal invasion	4 (1.0)

^a^ Four lesions were excluded due to missing information on margin status.

### 3.3 DB Rate of LPPC HSP

DB occurred in two patients (Table 3). In case 1, concurrent large lesions were resected using EMR. In case 2, a biopsy was taken from the concurrent advanced cecum cancer, and aspirin was discontinued 3 days before LPPC HSP and restarted on the day after the procedure. Both patients were hospitalized owing to hematochezia, on the same day as the resection in case 1 and on the following day in case 2. However, both patients were managed conservatively without endoscopic hemostasis or blood transfusion and discharged on the day after admission.

**TABLE 3:** Clinical information of patients with delayed bleeding.

	Age, sex	Antithrombotic agents	Resected polyps	Resected via LPPC HSP	Concurrent large lesions	Bleed timing	Blood transfusion	Endoscopic hemostasis
Case 1	46, male	None	12	A, 0‐Is, 10 mm	T, 0‐Is, 14 mm (EMR) S, 0‐Is, 14 mm (EMR)	Day 0	No	No
Case 2	73, male	Aspirin	4	A, 0‐IIa, 10 mm	Advanced cecum cancer, type 2, 40 mm	Day 1	No	No

Abbreviations: EMR, endoscopic mucosal resection; LPPC HSP, low‐power pure‐cut current hot snare polypectomy.

The DB rate was 0.6% for patients (2/339, 95% CI: 0.1%–2.1%) and 0.5% for lesions (2/410, 95% CI: 0.1%–1.8%) (Table 2). LPPC HSP was associated with a 71.4% reduction in the risk of DB per patient compared with the historical control (2.1%), with a power of 80.4% and a two‐sided significance level of 0.1.

## 4 Discussion

This retrospective study provided evidence supporting the safety profile of LPPC HSP. The DB rate of LPPC HSP for colorectal polyps sized 10–14 mm was 0.6%, lower than that of conventional HSP (2.1%). To the best of our knowledge, the DB rate of LPPC HSP is the lowest among the reported electrocautery‐based resection methods for colorectal polyps sized 10–14 mm.

We set the historical control DB rate as 2.1% based on a previous meta‐analysis of colorectal polyps sized 10–19 mm [4]. The size range of the lesions in the historical control was wider than that in our study, which might have led to overestimation of the DB rate. However, in a previous randomized controlled study, the DB rate of HSP/EMR for polyps sized 11–15 mm was 2.8% (7/249), higher than the 2.1% in the meta‐analysis [3]. Moreover, a more recent randomized controlled study revealed a DB rate of EMR for polyps 6–20 mm in size of 2.6% (7/267) [22]. Although their size range was wider than ours, the mean polyp size was 12.2 mm in their study, comparable to that in our study (11.5 mm). Therefore, we consider the historical control in this study reasonable.

In one retrospective study, the DB rate of CSP for polyps 10–14 mm in size was 0.8% (1/125) [23]. In the recent randomized controlled study, the DB rate of CSP/cold snare EMR for polyps 6–20 mm in size (mean size: 12.0 mm) was 1.0% (5/496) [22]. These results are comparable to the DB rate of LPPC HSP (0.6%) in our study, suggesting that LPPC HSP is as safe as CSP. A possible explanation for the low DB rate of LPPC HSP is the lower voltage of the electrosurgery unit, which may decrease the risk of heat injury to the deeper tissues. The authors of a previous study stated that DB may be related to blood‐vessel injuries caused by electrocautery in the submucosal layer [24]. In another study, the mucosal defect size increased within 1 day of HSP [25]. A study in an animal model demonstrated that thermal injury extended to the deep muscle layer in 0%, 13.4%, and 53.4% of the procedures for which pure‐cut, blended‐cut, and pure‐coagulation currents were applied [26]. Our experimental study revealed that the remaining submucosal layer was substantially thicker with LPPC HSP than with polypectomy with a blended‐cut current, suggesting that LPPC HSP may affect only the superficial parts of the submucosal layer, with reduced thermal damage [13]. In this context, LPPC HSP may yield a lower risk of blood‐vessel injury and result in a larger area of remaining tissue, leading to a lower risk of DB. This would explain why the risk of DB with LPPC HSP is lower than that with conventional HSP/EMR with blended‐ or coagulation‐cut currents. Furthermore, since the DB risk of underwater EMR has been shown to be comparable to that of conventional EMR [27], the DB risk with LPPC HSP is expected to be even lower.

Our results align with those of recent studies highlighting the resectability of adenomatous polyps sized 10–14 mm with LPPC HSP. The en bloc and R0 resection rates of LPPC were 94.9% and 86.7%, respectively, comparable with those in our phase I study (87.8% and 85.7%, respectively) and our recent study of LPPC HSP vs. EMR (98% and 92% vs. 96% and 90%) [13, 15]. In a randomized controlled trial of HSP vs. EMR, complete‐resection rates of 10–14‐mm polyps did not significantly differ (90% vs. 93%) [11]. Another randomized controlled trial has revealed similar complete‐resection rates of 10–14‐mm polyps via EMR and underwater EMR [28]. The slightly lower R0 resection rate in our study (86.7%) may be attributed to a higher proportion of lesions with indeterminate horizontal margins. While smaller resection specimens often lead to indeterminate margins, prior research suggests that this does not correlate with local recurrence, indicating limited clinical impact [29]. These results suggest that LPPC HSP yields a resectability comparable to that of conventional HSP, EMR, and underwater EMR for 10–14‐mm polyps. Considering its safety profile and resectability, LPPC HSP has the potential to supersede conventional resection methods such as EMR, underwater EMR, and CSP. LPPC HSP may also be feasible for patients taking antithrombotic agents, who have a higher risk of DB.

This study had several limitations. First, its retrospective nature posed a challenge. During the study period, the resection method for colorectal polyps sized 10–14 mm was at the discretion of the endoscopist, resulting in a selection bias. Second, the monitoring for the DB might have been insufficient. However, all patients were followed up at least once in an outpatient setting after the day of endoscopic resection, and only two patients made unscheduled visits to the physician owing to bleeding, as described in Table 3. As these patients had concurrent large lesions and could be conservatively managed without endoscopic hemostasis or blood transfusion, the bleeding might not have been due to LPPC HSP. In this context, the DB rate of LPPC HSP might have been overestimated. Third, only a prescribed, thin, hexagonal snare was used for LPPC HSP, enabling sharp and precise excision with minimal current application time. In contrast, using a thicker snare may necessitate a longer duration of electrocautery, resulting in greater thermal injury and a potentially increased risk of DB. Given the differing electrical intensities of snares with different diameters, our results may not be generalizable to other snare types. Finally, the type of electrosurgical generator used in each case is unknown. To overcome these issues, a prospective study is warranted to confirm our study outcomes.

In conclusion, our study demonstrated that the DB rate of LPPC HSP for nonpedunculated colorectal neoplasms sized 10–14 mm was 0.6% for patients and 0.5% for lesions, lower than reported rates of conventional HSP/EMR. A large, prospective study is warranted to confirm the safety of LPPC HSP.

## Author Contributions

**Kazunori Takada**, **Hidenori Kimura**, **Kinichi Hotta**, **Kenichiro Imai**, **Sayo Ito**, and **Yoshihiro Kishida** were responsible for the conception and design of the study. **Kazunori Takada** performed analysis and interpretation of the data with support from **Kenichiro Imai**, **Sayo Ito**, and **Yoshihiro Kishida**. **Kazunori Takada** drafted the article. **Hidenori Kimura**, **Yoichi Yamamoto**, **Masao Yoshida**, **Noboru Kawata**, **Junya Sato**, **Hirotoshi Ishiwatari**, **Hiroyuki Matsubayashi**, and **Hiroyuki Ono** performed critical revision of the article for important intellectual content. All authors read and approved the final version of the manuscript.

## Funding

The authors received no specific funding for this work.

## Conflicts of Interest

Kenichiro Imai has received research grants from KANEKA Corporation, the Japanese Foundation for research and promotion of endoscopy. Kenichiro Imai has received speaker honoraria from Olympus, Boston Scientific, and 3D matrix. The other authors declare no conflict of interest for this article.

## Ethics Statement

Approval of the research protocol by an Institutional Reviewer Board: This study was approved by the Ethics Committee of Shizuoka Cancer Center (T2022‐56‐2022‐1).

## Consent

Informed consent was waived owing to the retrospective nature of the study. Patients could opt out of participation in studies on the website of each institution.

## Data Availability

The patient data used to support the findings of this study are available from the corresponding author upon request.
